# Editorial: Chemoresistance and immunotherapy in solid tumors

**DOI:** 10.3389/fimmu.2026.2013215

**Published:** 2026-10-15

**Authors:** Junchao Xu

**Affiliations:** Department of Bioengineering, University of Pennsylvania, Philadelphia, PA, United States

**Keywords:** biomarkers, cancer immunotherapy, chemoresistance, drug delivery, longitudinal monitoring, tumor microenvironment

Chemotherapy and immune checkpoint blockade act on the same changing tissue. Cytotoxic treatment selects the cells that survive and alters the signals they send to neighboring immune and stromal cells. Immune pressure, in turn, shapes which tumor cells persist. The clinical task is to learn when this interaction can be altered, in which patients, and at what cost. Trials in advanced gastric and gastroesophageal cancer show that adding PD-1 blockade to chemotherapy can prolong survival, while the size of the benefit varies across patients (1, 2). This Research Topic set out to examine immune and stromal mechanisms of chemoresistance, find useful biomarkers, and improve combination treatment. Its papers address different parts of that problem, from tumor-cell survival to drug delivery, response, and toxicity. Most concern digestive cancers; their lessons for other solid tumors need testing.

## Tumor killing is one step

Three original studies examine ways in which cancer cells withstand or respond to treatment. In colorectal cancer, Wang et al. link IGFL1 expression to impaired lipophagy and tumor-cell growth. Their analyses also associate IGFL1 with immune infiltration and predicted treatment response, although those immune findings remain computational. In gastric cancer models, Li et al. find that p53-associated COX17 expression increases sensitivity to elesclomol-induced copper-associated cytotoxicity. The work includes endothelial-cell readouts but does not establish whether the induced tumor-cell death stimulates antitumor immunity. In ovarian cancer, Hong et al. combine molecular analyses and cell experiments to examine hydroxychloroquine and sulfasalazine as possible chemosensitizers. Their inflammatory and immune signatures warrant further testing; clinical benefit from the drug pair is not established.

These papers point to a distinction that should guide combination design. A drug may kill resistant cells without improving immune recognition. It may also change immune signaling without reaching enough malignant cells. Yang et al. review how cell-intrinsic defenses, immunogenic cell death, and the microenvironment can pull treatment in different directions. Cui et al. examine CDK4/6 inhibition as a means of changing both proliferation and immune function in gastrointestinal tumors. Together, these reviews suggest a practical question for each proposed combination: which step fails in this tumor—drug access, tumor-cell killing, antigen presentation, or immune-cell entry and function? The answer may change after treatment begins.

Access deserves particular attention. The prescribed dose is not the dose experienced by every tumor cell or immune cell. Tumor vessels, stroma, and lymphatic drainage can alter the distribution of cytotoxic drugs and antibodies. In mouse models, blocking tumor lymphangiogenesis increased intratumoral accumulation of doxorubicin formulations and anti-PD-L1 antibody (3). Lymphatic vessels also carry antigens and immune cells; changing their function may have competing effects on drug retention and immune priming. This is a reason to measure local exposure and immune function together, not evidence that the same approach will overcome resistance in patients.

## Clinical benefit has several dimensions

The clinical contributions show why a laboratory mechanism cannot, by itself, choose a regimen. In a retrospective cohort of locally advanced gastric cancer, Lu et al. associate routine blood measures with pathological response to neoadjuvant immunochemotherapy. Their analysis of treatment cycles raises a useful question, but a non-randomized subgroup cannot establish the best number of cycles. In gastroesophageal junction cancer, Zhang et al. report no significant increase in major postoperative complications after immunochemotherapy compared with chemotherapy in 120 propensity-matched pairs. This supports further study of surgical safety; it does not prove equal risk.

Benefit must also be weighed against immune toxicity. Yu et al. find more immune-related adverse events among patients with baseline antinuclear antibodies in a small retrospective cohort, without a clear increase in treatment discontinuation. Their findings support closer observation, not the use of autoantibodies to predict survival. A systematic review by Mi et al. reports responses to cadonilimab across solid tumors, but most included studies are single-arm trials and severe treatment-related events are common. Pooled response rates cannot tell us how much a regimen adds over a suitable comparator, or which patients should receive it. A marker that tracks response within a combination-treated group may be prognostic without predicting benefit from the immune agent. Establishing that distinction requires a comparator and a pre-specified test of whether treatment benefit differs by marker status.

Exceptional responses can expose questions that averages miss. Jin et al. describe durable control of AFP-producing metastatic gastric cancer with FOLFOX and serplulimab, with serum AFP tracking the response. Zhao et al. report prolonged survival after taxane-based chemo-immunotherapy in EGFR exon 20 insertion lung cancer, a setting in which checkpoint inhibition often has limited activity. Each case is worth investigating, but neither establishes a treatment rule. Tumor biology, prior therapy, and drug exposure could all help explain the outcome.

## Measure change, then test decisions

Resistance develops over time, whereas many proposed biomarkers are measured once. Li et al. describe how circulating tumor DNA, circulating tumor cells, and extracellular vesicles might track emerging resistance. Blood sampling can reveal molecular change, but it cannot on its own show whether drugs and immune cells reach a lesion or whether nearby cells suppress an immune response. Serial blood measurements should therefore be paired, where feasible, with tissue, imaging, and treatment-exposure data.

The next studies should specify the decision a marker is meant to change before testing it. For example, if an early blood signal suggests resistance, would switching treatment improve survival, or merely change a laboratory result? If chemotherapy increases tumor-cell death, do paired samples show more antigen presentation and functional T cells, and does that change last? If a combination appears active, can patients remain on it without disproportionate harm? An informative prospective study would collect plasma before and during treatment, sample tumor tissue at defined points, and assess drug exposure, immune state, response, and toxicity together. A marker-guided treatment change would then need a controlled test. None of the original studies in this collection provides that test. This distinction matters when reading evidence from cell lines, animal models, observational cohorts, and individual cases.

This Research Topic brings these levels of evidence into view. It offers candidate mechanisms, tools for monitoring, and clinical observations across several cancers. Its lasting contribution will depend on whether future studies connect tumor-cell response, local drug access, immune function, and patient outcomes in the same patients. That connection is the route from a plausible combination to a useful one.
